# Single-Atom Catalysts (SACs) for High-Efficiency Water Electrolysis: A Comprehensive Review

**DOI:** 10.3390/ma19163375

**Published:** 2026-08-07

**Authors:** Farhan Akhtar, Wajid Ali, Muhammad Saqib, Syed Adil Sardar, Tabinda Shabir, Muhammad Awais, Samina Karim, Woo Young Kim

**Affiliations:** 1 Department of Electronic Engineering, Jeju National University, Jeju 63243, Republic of Korea; 2 Ajou Energy Science Research Center, Ajou University, Suwon 16499, Republic of Korea

**Keywords:** single-atom catalysts, water splitting, electrocatalysis, hydrogen evolution reaction (HER), oxygen evolution reaction (OER), coordination engineering

## Abstract

Hydrogen produced through electrochemical water splitting is considered one of the most promising energy carriers for achieving a sustainable and carbon-neutral future. However, the practical implementation of water electrolysis remains limited by the sluggish kinetics of the hydrogen evolution reaction (HER) and oxygen evolution reaction (OER), as well as the high cost and limited availability of conventional noble-metal catalysts. Single-atom catalysts (SACs), which feature isolated metal atoms anchored on suitable supports, have emerged as an attractive class of electrocatalysts owing to their nearly complete atomic utilization, well-defined active sites, and tunable electronic structures. This review provides a comprehensive overview of recent advances in SACs for electrochemical water splitting. The fundamental mechanisms of HER and OER are first discussed, followed by the influence of the unique electronic structure, coordination environment, and metal–support interactions on catalytic performance. Various bottom-up and top-down synthesis strategies, together with advanced characterization techniques for identifying atomically dispersed active sites and elucidating structure–activity relationships, are systematically summarized. Furthermore, recent progress in noble-metal, non-noble-metal, and dual-atom catalysts is critically reviewed, with emphasis on their roles in regulating electronic structure, reaction intermediate adsorption, catalytic activity, HER/OER kinetics, and long-term stability. Finally, the remaining challenges and future perspectives for the scalable and practical application of SACs in water electrolysis are discussed. Overall, this review highlights the potential of SACs to maximize metal utilization while maintaining high electrocatalytic performance and provides valuable insights for the rational design of next-generation electrocatalysts for sustainable hydrogen production.

## 1. Introduction

The rapid industrialization of modern society and the extensive consumption of fossil fuels have led to a substantial increase in atmospheric carbon dioxide (CO_2_) concentration from approximately 280 ppm in the pre-industrial era to current levels exceeding 425 ppm. According to the NOAA Global Monitoring Laboratory, the daily average atmospheric CO_2_ concentration at the Mauna Loa Observatory has reached approximately 427.8 ppm, highlighting the continued challenge of mitigating greenhouse gas emissions and their associated impacts, including global warming, climate change, and rising sea levels. These environmental concerns have accelerated the global transition toward sustainable, renewable, and carbon-neutral energy systems [1,2]. Among the various clean energy carriers, green hydrogen (H_2_) has attracted considerable attention owing to its high gravimetric energy density (142 MJ kg^−1^), zero-carbon combustion, and its essential role in industrial processes such as ammonia synthesis and chemical manufacturing [3]. However, global hydrogen production remains overwhelmingly dependent on fossil-fuel-based routes. Recent reports from the International Energy Agency (IEA) indicate that approximately 99% of global hydrogen production still relies on carbon-intensive, unabated fossil fuel pathways, including steam methane reforming and coal gasification, while low-emission hydrogen contributes less than 1% of total production. Consequently, electrochemical water splitting, powered by renewable energy sources such as solar and wind, has emerged as one of the most promising technologies for sustainable, high-purity hydrogen production [2].

Electrochemical water splitting consists of two coupled half-reactions: the hydrogen evolution reaction (HER) at the cathode and the oxygen evolution reaction (OER) at the anode [4]. Although the overall reaction has a theoretical equilibrium potential of 1.23 V [5], practical electrolyzers generally require much higher operating voltages (typically 1.8–2.0 V) because of the sluggish kinetics of both half-reactions, particularly the four-electron OER, which represents the major kinetic bottleneck of the process [6]. At present, noble metal-based electrocatalysts, including Pt for HER and Ir- or Ru-based oxides for OER, remain the state-of-the-art catalysts owing to their excellent catalytic activity [7]. Nevertheless, their high cost, limited natural abundance, and insufficient long-term durability under harsh electrochemical conditions severely restrict large-scale commercial deployment [8].

To reduce the dependence on noble metals, extensive efforts have been devoted to developing alternative electrocatalysts based on earth-abundant transition metals. Representative materials include transition metals other than noble metals, metal oxides or hydroxides [9,10], metal phosphides [11], metal chalcogenides [12], metal nitrides [13], metal–organic frameworks (MOFs), and carbon/carbonitride-based and metal-free electrocatalysts [14,15,16,17,18]. Although many of these materials exhibit encouraging electrocatalytic performance, their intrinsic activity and reaction kinetics generally remain inferior to those of noble metal catalysts, particularly under demanding operating conditions [19]. Consequently, designing catalysts that simultaneously achieve high activity, excellent stability, and low cost remains one of the central challenges in water electrolysis research.

In recent years, single-atom catalysts (SACs) have emerged as a transformative class of electrocatalysts capable of addressing many of these limitations [20,21]. First proposed by Zhang and co-workers in 2011, SACs consist of isolated metal atoms atomically dispersed and strongly anchored on suitable solid supports [22,23]. Unlike conventional nanoparticle catalysts, every metal atom in SACs can potentially function as an active catalytic site, enabling nearly 100% atomic utilization efficiency and substantially reducing the consumption of precious metals [24]. Moreover, the unsaturated coordination environment and quantum size effects associated with isolated atoms generate unique electronic structures that can significantly improve intrinsic catalytic activity [25]. Another defining feature of SACs is the strong metal–support interaction (MSI), through which the electronic structure, oxidation state, and coordination environment of the isolated metal center can be precisely regulated to optimize the adsorption and desorption of reaction intermediates [26]. As a result, SACs effectively bridge the advantages of homogeneous catalysis, including well-defined active sites and high selectivity [27,28], with the excellent stability and recyclability of heterogeneous catalysts [29].

The performance of SACs is closely governed by the nature of the support material. Beyond stabilizing isolated metal atoms against aggregation driven by their high surface free energy [30], supports actively regulate the local electronic structure of the metal centers and often participate directly in catalytic reactions through synergistic interactions. Carbonaceous materials such as porous carbon, graphene, and carbon nanotubes [31,32], transition metal compounds including oxides, hydroxides, phosphides, and chalcogenides [33], MXenes [34], and metal–organic frameworks (MOFs) [35] have all demonstrated excellent capabilities for anchoring atomically dispersed active sites. More recently, sustainable support materials derived from biomass and waste resources have attracted increasing attention as environmentally friendly alternatives for SAC fabrication [36]. In parallel, catalyst design has progressed beyond isolated single-metal centers toward bimetallic and dual-atom catalysts (DACs), where synergistic interactions between adjacent metal atoms can overcome the scaling relationship limitations associated with conventional single-site catalysts and further enhance electrocatalytic performance [37].

This review provides a comprehensive overview of the recent progress in transition metal single-atom catalysts (TMSACs) for electrochemical water splitting. It first discusses the fundamental reaction mechanisms governing HER and OER and highlights how the unique electronic structures and coordination environments of SACs influence catalytic activity. Subsequently, the major synthesis strategies, including bottom-up and top-down approaches, together with advanced characterization techniques for identifying atomically dispersed active sites, are systematically summarized. The review then examines the critical roles of support engineering, coordination environment regulation, and metal–support interactions in determining catalytic performance, followed by a discussion of recent advances in noble-metal, non-noble-metal, and dual-atom catalyst systems for efficient water splitting. Finally, the key challenges associated with scalable synthesis, high metal loading, long-term stability, mechanistic understanding, and practical implementation are discussed, together with future perspectives for advancing SACs toward commercially viable hydrogen production technologies.

## 2. Fundamentals and Catalytic Mechanisms of Water Electrolysis

The electrochemical decomposition of water (2H_2_O → 2H_2_ + O_2_) is the fundamental reaction underlying sustainable hydrogen production and has attracted considerable attention as a clean pathway for generating high-purity green hydrogen [38]. As discussed above, practical water electrolysis generally requires cell voltages exceeding the thermodynamic potential because of kinetic overpotentials [39]. These overpotentials primarily arise from the sluggish kinetics of the Hydrogen Evolution Reaction (HER) occurring at the cathode and the Oxygen Evolution Reaction (OER) occurring at the anode [40]. Single-atom catalysts (SACs) have emerged as promising electrocatalysts for water splitting because they maximize metal atom utilization, provide well-defined and uniform active sites, and allow for precise tuning of the electronic structure through coordination engineering and metal–support interactions [41]. These characteristics enable the optimization of adsorption and desorption energies of key reaction intermediates, thereby lowering activation barriers and reducing the overpotentials required for efficient HER and OER.

### 2.1. Hydrogen Evolution Reaction (HER) Mechanisms

**Figure 1:**
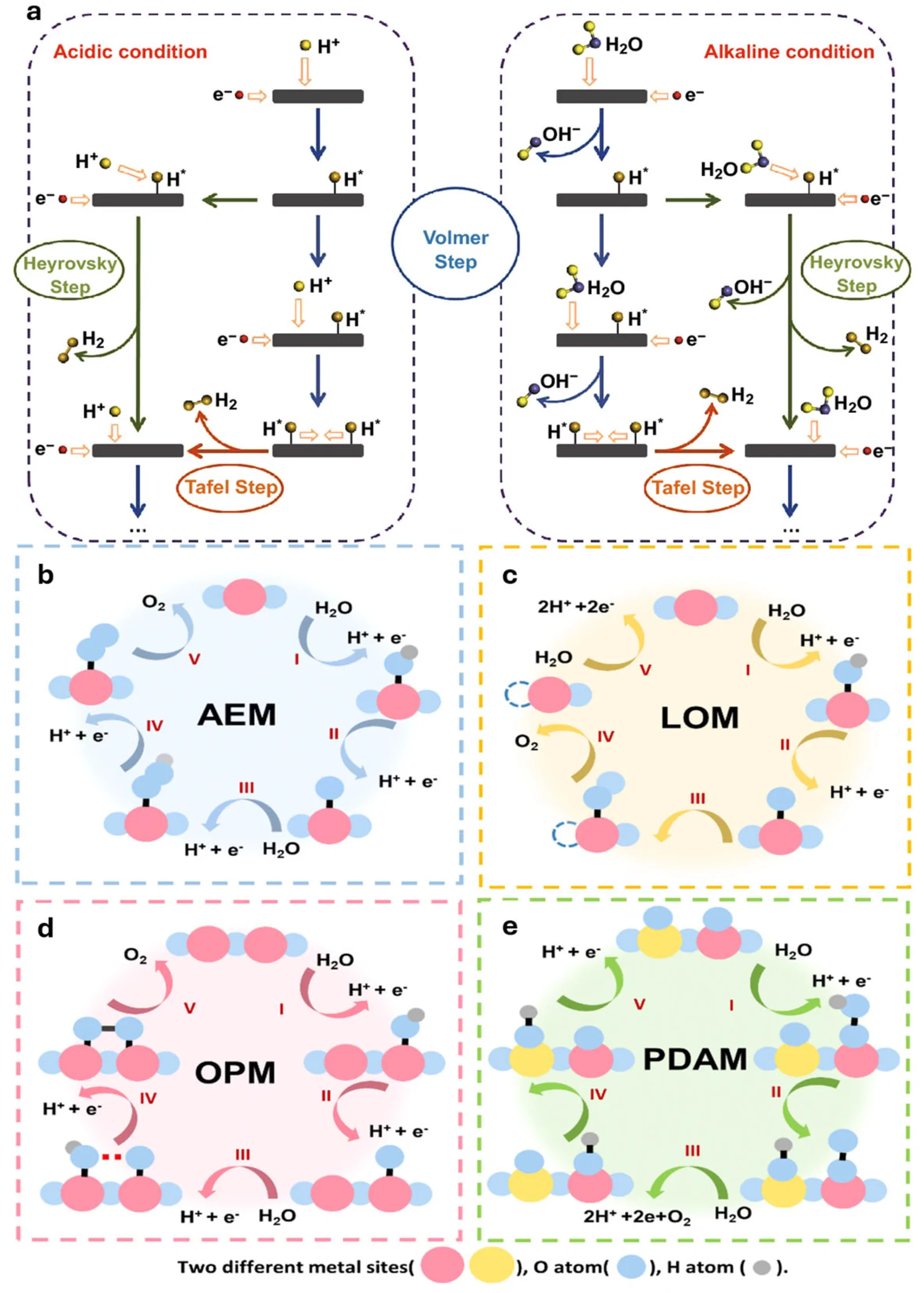
Fundamental reaction mechanisms involved in water electrolysis. (**a**) Schematic pathways of the hydrogen evolution reaction (HER) in acidic and alkaline electrolytes, illustrating the Volmer, Heyrovsky, and Tafel steps. (**b**–**e**) Representative oxygen evolution reaction (OER) pathways, including the adsorbate evolution mechanism (AEM), lattice oxygen mechanism (LOM), oxide path mechanism (OPM), and proton donor–acceptor mechanism (PDAM), highlighting the distinct routes for O–O bond formation during water oxidation. Adapted from Refs. [43,60].

Hydrogen Evolution Reaction (HER) is a two-electron electrochemical process that converts protons or water molecules into molecular hydrogen through a sequence of elementary proton-coupled electron-transfer steps [42]. As illustrated in Figure 1a [43], HER proceeds through the Volmer, Heyrovsky, and Tafel steps, while the dominant reaction pathway depends on the electrolyte pH and the nature of the catalyst surface [44]. Since the proton source differs between acidic and alkaline electrolytes, the reaction kinetics and rate-determining steps also vary considerably.

#### 2.1.1. Reaction Pathways in Acidic and Alkaline Media

As shown in Figure 1a, HER in acidic electrolytes proceeds through the direct reduction of hydronium ions (H_3_O^+^), following either the Volmer–Heyrovsky or the Volmer–Tafel pathway [45]. The elementary reaction steps are summarized below [46].

Volmer step (electrochemical discharge):

H_3_O^+^(aq) + e^−^ + * → H* + H_2_O(l)

where * denotes an active catalytic site and H* represents adsorbed hydrogen.

Heyrovsky step (electrochemical desorption):

H* + H_3_O^+^(aq) + e^−^ → H_2_(g) + H_2_O(l) + *

Tafel step (chemical recombination):

2H* → H_2_(g) + 2*

Depending on the catalyst surface and reaction kinetics, hydrogen evolution proceeds through either the Volmer–Heyrovsky pathway or the Volmer–Tafel pathway, both of which ultimately produce molecular hydrogen.

In alkaline electrolytes, the reaction mechanism remains fundamentally similar but becomes kinetically slower because water rather than hydronium ions serves as the proton source [47]. Consequently, the initial Volmer step requires the dissociation of water molecules, involving cleavage of the strong O–H bond before hydrogen adsorption can occur. This additional energy requirement is largely responsible for the inferior HER kinetics in alkaline media [48].

The elementary reaction steps in alkaline electrolytes are

Volmer step:

H_2_O(l) + e^−^ + * → H* + OH^−^(aq)

Heyrovsky step:

H* + H_2_O(l) + e^−^ → H_2_(g) + OH^−^(aq) + *

Tafel step:

2H* → H_2_(g) + 2*

As illustrated in Figure 1a, the acidic and alkaline HER pathways share the same hydrogen evolution routes after H* formation. However, the additional water dissociation step required in alkaline media introduces a larger kinetic barrier, making catalyst design strategies that promote water activation particularly important for efficient alkaline HER.

#### 2.1.2. Key Kinetic Parameters and Descriptors

The intrinsic HER activity of electrocatalysts is commonly evaluated using several kinetic descriptors that quantify the thermodynamics and kinetics of hydrogen evolution.

- Hydrogen Adsorption Free Energy (ΔGH*): According to the Sabatier principle, an ideal HER catalyst should exhibit a near-thermoneutral hydrogen adsorption free energy (ΔGH*) close to zero [49]. If ΔGH* is too negative, hydrogen binds too strongly to the catalyst surface, which impedes H_2_ desorption; if it is too positive, hydrogen adsorption becomes unfavorable and proton reduction is kinetically hindered. Thus, tuning ΔGH* toward zero is a key criterion for balancing hydrogen adsorption and desorption during HER. Pt-based single-atom catalysts remain the benchmark because their ΔGH* values are close to thermoneutral [50].
- Tafel Slopes: The Tafel slope (b) provides valuable insight into HER kinetics and helps identify the rate-determining step (RDS) [51]. Under ideal Butler–Volmer kinetics with a charge transfer coefficient (α) of approximately 0.5, Tafel slopes of ~120, ~40, and ~30 mV dec^−1^ are commonly associated with the Volmer, Heyrovsky, and Tafel rate-determining steps, respectively. However, these values should be interpreted with caution, as experimentally measured Tafel slopes can be influenced by factors such as the selected potential window, surface coverage effects, mass transport limitations, catalyst reconstruction, and deviations from ideal kinetic assumptions. Therefore, Tafel analysis should be complemented with additional electrochemical and mechanistic evidence when identifying the HER pathway.
- Exchange Current Density (j_0_): The exchange current density (j_0_) represents the intrinsic catalytic activity at equilibrium. Higher j_0_ values indicate faster charge-transfer kinetics and lower activation barriers for HER, reflecting superior electrocatalytic performance independent of externally applied overpotential.

#### 2.1.3. Structural Engineering of SACs for HER

Beyond intrinsic reaction kinetics, the catalytic performance of SACs is strongly governed by the local coordination environment of the isolated metal atoms and their interactions with the supporting substrate, commonly referred to as metal–support interactions (MSIs) [52]. Rational engineering of these structural features enables modulation of the electronic structure of the active sites and optimization of hydrogen adsorption, water activation, and charge-transfer processes.

- Electronic Modulation: In carbon-supported SACs, isolated metal atoms are frequently coordinated with nitrogen atoms to form M–Nx active sites, where the coordination environment significantly influences the electronic structure of the metal center [22]. Variations in the coordination number, such as M–N_2_ or M–N_3_ configurations, alter the d-band center and redistribute the local charge density, thereby optimizing hydrogen adsorption and facilitating HER [53]. Low-coordination structures generally expose more unsaturated active sites and often exhibit higher intrinsic catalytic activity than fully coordinated configurations.
- Support-Mediated Synergy: The support not only anchors isolated metal atoms but also actively participates in the catalytic process. Atomically thin materials (ATMs), including MoS_2_ and MXenes, provide abundant vacancies, defects, and unsaturated surface atoms that strongly stabilize single atoms while simultaneously promoting water dissociation, particularly under alkaline conditions [54]. In addition, Single-Atom Alloys (SAAs), where isolated catalytic atoms are embedded within a metallic host, can modify the adsorption energies of reaction intermediates and partially overcome conventional scaling relationships, enabling more efficient hydrogen evolution through cooperative substrate activation and H–H coupling [55].
- Hydrogen Spillover: Hydrogen spillover is another important phenomenon contributing to enhanced HER activity. Following hydrogen adsorption on the isolated metal center, adsorbed hydrogen (H*) can migrate onto the support surface, thereby regenerating active sites for subsequent proton reduction while increasing the effective surface coverage of reactive hydrogen species [56]. This synergistic interaction between isolated metal atoms and the support improves catalyst utilization, accelerates hydrogen evolution kinetics, and contributes to the excellent HER performance observed in many SAC systems.

### 2.2. Oxygen Evolution Reaction (OER) Mechanisms

The Oxygen Evolution Reaction (OER) is the anodic half-reaction of water electrolysis and is generally regarded as the primary kinetic bottleneck for overall water splitting because it involves a complex four-electron/four-proton transfer process accompanied by O–H bond cleavage and O–O bond formation [57]. Although the thermodynamic equilibrium potential for OER is 1.23 V, practical electrolyzers typically require overpotentials exceeding 300–400 mV to achieve industrially relevant current densities due to the sluggish reaction kinetics [58,59]. Consequently, the development of highly active and durable OER electrocatalysts is essential for improving the overall efficiency of water electrolysis.

Single-atom catalysts (SACs) have emerged as promising OER electrocatalysts because their atomically dispersed metal centers possess well-defined coordination environments that enable precise regulation of adsorption energies for oxygen-containing intermediates. Furthermore, the strong interaction between isolated metal atoms and their supports facilitates charge redistribution, optimizes reaction energetics, and improves catalytic stability under oxidative operating conditions.

#### 2.2.1. Primary OER Pathways: Adsorbate Evolution Mechanism (AEM) and Lattice Oxygen Mechanism (LOM)

As illustrated in Figure 1b,c [60], two principal mechanisms have been proposed to describe OER on SACs and other transition-metal catalysts: the conventional Adsorbate Evolution Mechanism (AEM) and the Lattice Oxygen Mechanism (LOM) [61,62,63]. While both pathways ultimately generate molecular oxygen, they differ fundamentally in the origin of the oxygen atoms participating in O–O bond formation.

Adsorbate Evolution Mechanism (AEM):

The AEM (Figure 1b) is the most widely accepted OER pathway, in which the isolated metal atom serves as the catalytic center for the sequential adsorption and conversion of oxygen-containing intermediates [64]. The reaction proceeds through the stepwise formation of OH*, O*, and OOH* species before the release of O_2_.

Acidic Media [65]:

M* + H_2_O(l) → M–OH* + H^+^(aq) + e^−^

M–OH* → M–O* + H^+^(aq) + e^−^

M–O* + H_2_O(l) → M–OOH* + H^+^(aq) + e^−^

M–OOH* → M* + O_2_(g) + H^+^(aq) + e^−^

Alkaline Media [66]:

M* + OH^−^(aq) → M–OH* + e^−^

M–OH* + OH^−^(aq) → M–O* + H_2_O(l) + e^−^

M–O* + OH^−^(aq) → M–OOH* + e^−^

M–OOH* + OH^−^(aq) → M* + O_2_(g) + H_2_O(l) + e^−^

For SACs, the adsorption strength of OH*, O*, and OOH* is highly sensitive to the coordination environment of the isolated metal atom. Rational tuning of the electronic structure through metal–support interactions or coordination engineering enables optimization of these adsorption energies and consequently lowers the OER overpotential. A major limitation of the AEM is the intrinsic linear scaling relationship between the adsorption energies of OH*, O*, and OOH* intermediates, which imposes a theoretical minimum overpotential that is difficult to overcome even with highly active catalysts [67]. Therefore, considerable efforts in SAC design focus on regulating the electronic structure of isolated active sites to partially break these scaling relationships.

Lattice Oxygen Mechanism (LOM):

Unlike the AEM, the LOM (Figure 1c) involves the direct participation of lattice oxygen atoms from the catalyst framework in O–O bond formation [68]. In this pathway, an adsorbed oxygen intermediate couples with a neighboring lattice oxygen atom to generate O_2_, leaving behind an oxygen vacancy that is subsequently replenished by oxygen species from the electrolyte [69]. Because the O–O bond is formed through lattice oxygen rather than solely through adsorbed intermediates, the LOM can partially circumvent the scaling relationship that limits the AEM, thereby providing a pathway toward significantly lower theoretical overpotentials [70]. For many oxide-supported SACs, particularly those possessing highly covalent metal–oxygen bonds and abundant oxygen vacancies, lattice oxygen participation has been proposed to contribute substantially to the enhanced OER activity.

Despite these kinetic advantages, continuous formation and replenishment of oxygen vacancies may gradually destabilize the catalyst framework, resulting in lattice reconstruction, structural degradation, or metal leaching during prolonged electrolysis [71]. Therefore, balancing lattice oxygen activity with structural stability remains an important challenge in the rational design of highly durable SACs for OER.

#### 2.2.2. Emerging and Alternative Mechanisms

In addition to the conventional AEM and LOM pathways, several alternative mechanisms have been proposed to explain the exceptional OER activity of advanced electrocatalysts, particularly SACs with well-defined atomic coordination environments and strong metal–support interactions. As illustrated in Figure 1d,e [60], these mechanisms provide alternative routes for O–O bond formation and proton-coupled electron transfer, offering new opportunities to overcome the intrinsic kinetic limitations of conventional OER pathways.

Oxide Path Mechanism (OPM):

The Oxide Path Mechanism (OPM) (Figure 1d) has been proposed as an alternative OER pathway in which oxygen evolution proceeds through the direct coupling of adjacent oxygen radicals (O*) without forming the conventional OOH* intermediate [69]. By bypassing this energetically demanding intermediate, the OPM can potentially reduce the overall reaction barrier and improve catalytic kinetics. However, this mechanism generally requires precisely arranged neighboring active sites capable of simultaneously stabilizing adjacent oxygen species, making it particularly relevant to dual-atom catalysts (DACs) and closely coupled multi-atom active centers [72]. Although the OPM is less frequently reported for isolated SACs, understanding this pathway provides valuable insight into the evolution from single-site to multi-site catalytic systems for water oxidation.

Proton Donor–Acceptor Mechanism (PDAM):

As shown in Figure 1e, the Proton Donor–Acceptor Mechanism (PDAM) introduces an alternative proton-transfer pathway during OER, in which neighboring proton donor and acceptor sites cooperatively facilitate proton-coupled electron transfer. Such proton shuttling can lower the activation energy of elementary reaction steps and accelerate O–O bond formation. For SACs, the well-defined coordination environment and the presence of heteroatom ligands or hydroxylated support surfaces can provide favorable proton-transfer channels, making PDAM a promising mechanism for enhancing OER kinetics.

Beyond the mechanisms illustrated in Figure 1, several additional OER pathways have also been proposed, including the oxygen vacancy-site mechanism (OVSM) and modified adsorbate evolution pathways involving unconventional intermediates (e.g., syn-OHOH and anti-OHOH conformers) on certain carbon-supported SACs. However, these mechanisms remain relatively system-specific and are currently less established than the AEM, LOM, OPM, and PDAM pathways [73,74]. Overall, the coexistence of multiple OER mechanisms demonstrates that the catalytic behavior of SACs is strongly influenced by the electronic structure of the isolated metal center, its coordination environment, and the physicochemical properties of the support. Rational tuning of these structural features enables the preferential activation of specific reaction pathways, providing an effective strategy for reducing overpotentials while simultaneously improving catalytic activity, selectivity, and long-term stability in water-splitting electrocatalysis.

#### 2.2.3. The Role of SAC Coordination in OER Kinetics

The OER activity of single-atom catalysts (SACs) is largely governed by the adsorption strength of oxygen-containing intermediates on the isolated metal center, which is strongly influenced by its electronic structure and coordination environment [67]. Rational tuning of these factors enables optimization of the reaction pathway and reduction in the overall energy barrier for OER.

Active Site Identification:

In many Ru- and Ir-based SACs, the conversion of O* to OOH* is identified as the rate-determining step (RDS) [75]. Consequently, catalytic performance depends strongly on the ability of the coordinated metal center to optimize the adsorption energies of these intermediates while minimizing the associated energy barrier.

Electronic Modulation:

Strong electronic interactions between the single atom and the support play a crucial role in regulating catalytic activity. For example, electron transfer from supports such as NiO or MnO_2_ to isolated Ir atoms can stabilize high-valence Ir species, thereby optimizing the adsorption strength of key intermediates such as O* and OH* and promoting more efficient oxygen evolution [76]. More generally, changes in coordination number and ligand identity modify the local electronic structure of the active site, resulting in catalyst-dependent reaction pathways and OER activities.

Overall, the catalytic behavior of SACs is determined by the intimate relationship between the coordination environment, electronic structure, and intermediate adsorption. Detailed coordination engineering strategies used to tailor these properties are discussed in Section 4.4.

### 2.3. Computational Design of Single-Atom Catalysts

Density functional theory (DFT) has become an indispensable tool for the rational design of single-atom catalysts (SACs) because it enables atomic-scale understanding of the relationship between the electronic structure of isolated metal centers and their electrocatalytic activity [77]. In contrast to conventional trial-and-error experimentation, DFT provides quantitative insight into reaction energetics, charge distribution, adsorption configurations, and the influence of the coordination environment, thereby guiding the selection of promising catalyst compositions before experimental synthesis [78]. Consequently, computational studies now play a central role in identifying active sites, optimizing coordination environments, and predicting catalytic performance for water electrolysis [79].

For the hydrogen evolution reaction (HER), catalytic activity is commonly evaluated using the Gibbs free energy of hydrogen adsorption (ΔG_H*_) [80]. This descriptor is typically calculated using the Computational Hydrogen Electrode (CHE) model, which relates proton–electron transfer to the chemical potential of one-half of a hydrogen molecule under standard conditions. An ideal HER catalyst exhibits a ΔG_H*_ value close to zero, indicating a balance between hydrogen adsorption and desorption. Excessively negative values result in strong hydrogen binding and hinder H_2_ release, whereas excessively positive values indicate insufficient hydrogen adsorption. Therefore, ΔG_H*_ has become one of the most widely used computational descriptors for screening HER electrocatalysts [81].

Similarly, DFT calculations are extensively employed to investigate the oxygen evolution reaction (OER) by constructing free-energy diagrams for the elementary reaction intermediates, including OH*, O*, and OOH*. The Gibbs free-energy changes associated with these intermediates enable identification of the potential-determining step and estimation of the theoretical overpotential [82]. Such analyses provide valuable mechanistic insight into how variations in the metal center, coordination environment, defects, and metal–support interactions influence reaction energetics and catalytic performance.

Another important concept in computational catalyst design is the d-band center theory [83]. The position of the metal d-band relative to the Fermi level governs the interaction strength between the catalyst surface and adsorbed reaction intermediates. An upward shift in the d-band center generally strengthens adsorption, whereas a downward shift weakens the interaction. Rational modulation of the d-band center through coordination engineering, heteroatom doping, defect creation, or metal–support interactions therefore enables optimization of intermediate adsorption energies and enhancement of HER and OER kinetics [84]. This concept also provides the theoretical basis for the volcano relationship, in which maximum catalytic activity is achieved at an optimum adsorption strength rather than at either extreme of excessively strong or weak adsorption.

Although DFT has greatly accelerated catalyst discovery, several limitations should be recognized. Most calculations are performed on idealized structural models under equilibrium conditions and therefore cannot fully capture solvent effects, electric-field influences, dynamic surface reconstruction, or changes in coordination environment that occur during practical electrochemical operation [85]. In recent years, machine learning (ML) combined with high-throughput DFT calculations has emerged as a powerful strategy for accelerating catalyst discovery by rapidly screening large compositional spaces and identifying key structure–activity descriptors [86]. Nevertheless, computational predictions must ultimately be validated through experimental characterization and operando studies to establish reliable structure–activity relationships for practical water electrolysis.

## 3. Advanced Synthetic Strategies for SACs

The synthesis of high-quality Single-Atom Catalysts (SACs) requires methods that can achieve uniform atomic dispersion and robust anchoring onto support matrices. These strategies are broadly classified into bottom-up and top-down approaches based on the state of the metal precursors.

### 3.1. Bottom-Up Strategies

Bottom-up strategies construct SACs by anchoring metal ions or molecular precursors onto suitable substrates followed by thermal, electrochemical, or photochemical conversion into isolated active sites. These methods offer precise control over metal dispersion and coordination environments, making them among the most widely adopted approaches for SAC synthesis.

#### 3.1.1. High-Temperature Pyrolysis

**Figure 2:**
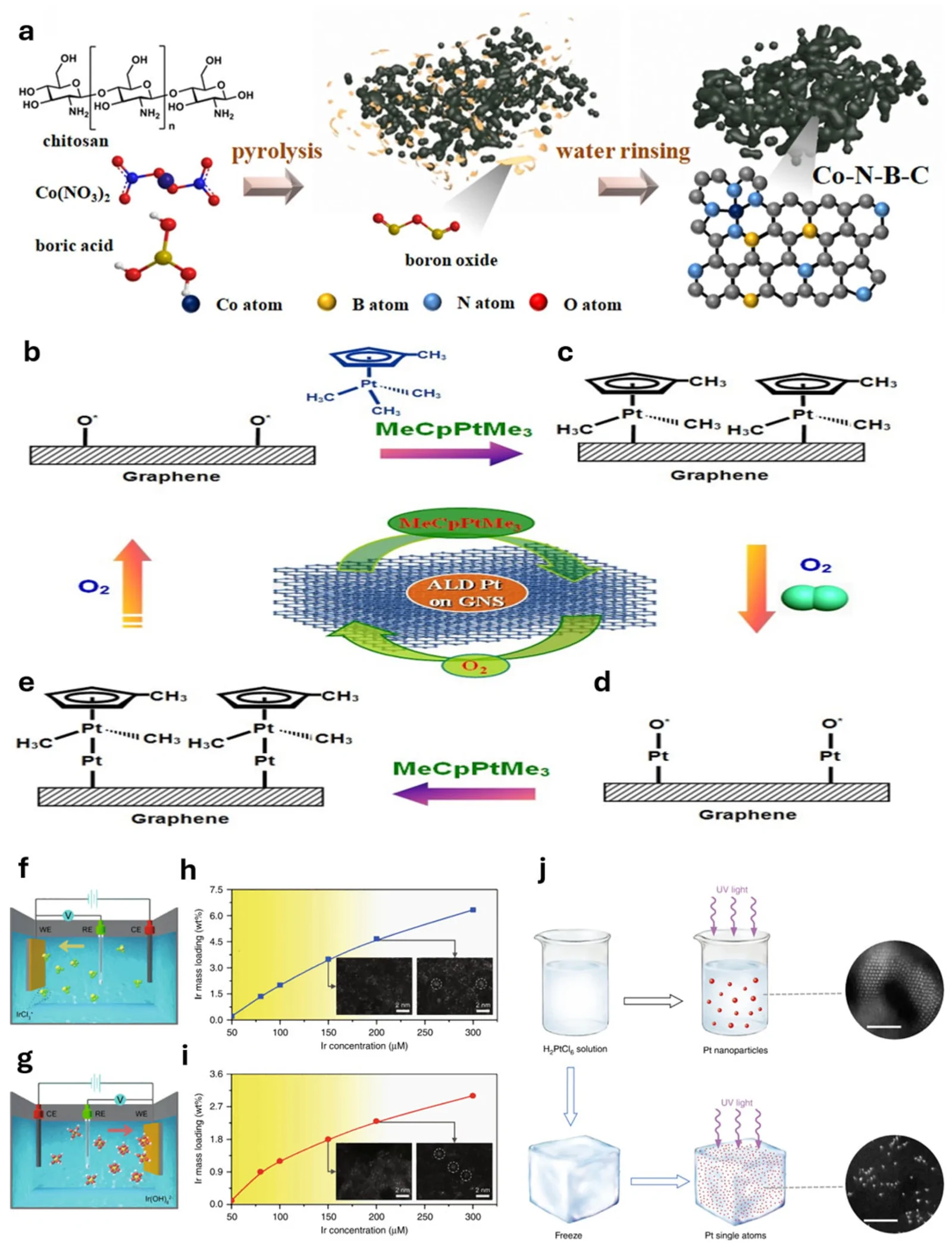
Representative bottom-up synthesis strategies for single-atom catalysts. (**a**) Schematic illustration of the high-temperature pyrolysis route for preparing Co–N–B–C, where chitosan, cobalt salt, and H_3_BO_3_ are pyrolyzed to generate atomically dispersed Co sites within a porous heteroatom-doped carbon matrix. (**b**–**e**) Atomic layer deposition (ALD) process for the controlled deposition of Pt atoms on graphene nanosheets through sequential precursor and oxygen exposures, enabling atomic-level regulation of Pt loading. (**f**–**i**) Electrochemical deposition of Ir species via cathodic and anodic routes, showing the influence of precursor concentration on Ir loading and the transition from isolated atoms to clusters. (**j**) Iced-photochemical reduction strategy, in which freezing suppresses atomic migration and nucleation, enabling the formation of isolated Pt single atoms rather than nanoparticles. Adapted from Refs. [88,90,93,95].

This is the most common approach for fabricating carbon-supported SACs [87]. It involves the thermal decomposition of metal-containing precursors, such as MOFs, polymers, or metal complexes, under inert or reactive atmospheres to generate atomically dispersed metal sites embedded within heteroatom-doped carbon matrices [20]. As illustrated in Figure 2a, pyrolysis of chitosan, cobalt salts, and boric acid produces Co–N–B–C with abundant pores and carbon-edge defects that effectively stabilize isolated Co atoms. This method is attractive because of its scalability, tunable coordination structures, and potential for high metal loading [88].

#### 3.1.2. Atomic Layer Deposition (ALD)

ALD utilizes self-limiting surface reactions to deposit metal species layer-by-layer from the vapor phase with atomic-level precision [89]. This gas-phase strategy allows metal atoms to penetrate deep into porous substrates, maximizing active site loading. As shown in Figure 2b–e, oxygen-containing functional groups on graphene first react with the Pt precursor (MeCpPtMe_3_), followed by oxygen exposure to regenerate active surface sites and complete one ALD cycle. Repetition of these cycles enables precise control of Pt loading while maintaining atomic dispersion [90]. Owing to its exceptional precision, ALD is also highly suitable for constructing dual-atom and bimetallic catalytic sites [91].

#### 3.1.3. Electrochemical Deposition

Electrochemical deposition utilizes an applied potential to reduce metal ions onto conductive supports under mild conditions without requiring additional reducing agents. It offers site-specific growth and the ability to control loading by adjusting potential, current density, or scanning cycles [92]. Figure 2f–i presents cathodic and anodic deposition pathways for Ir species, demonstrating how metal loading and dispersion can be regulated by electrolyte concentration and deposition conditions. The transition from isolated atoms to clusters with increasing precursor concentration highlights the importance of optimizing deposition parameters for achieving stable single-atom configurations [93].

#### 3.1.4. Wet-Chemical Methods (Impregnation & Co-Precipitation)

Wet-chemical methods involve the adsorption or co-precipitation of metal precursors with support materials in solution, utilizing surface defects, vacancies, or functional groups as anchoring sites [94]. These approaches are straightforward, cost-effective, and readily scalable, making them attractive for large-scale SAC production. However, achieving uniform atomic dispersion often requires careful control of precursor concentration and post-treatment conditions.

#### 3.1.5. Photochemical Reduction

Photochemical reduction employs light irradiation to convert metal ions into isolated metal atoms on suitable supports. An advanced variant, known as iced-photochemical reduction, utilizes a frozen precursor solution to suppress atomic diffusion and nucleation. As shown in Figure 2j, the ice lattice acts as a natural confinement medium that prevents aggregation during UV irradiation, enabling the formation of atomically dispersed Pt sites instead of Pt nanoparticles [95].

### 3.2. Top-Down Strategies

Top-down approaches utilize bulk metals or nanoparticles as precursors, breaking metal–metal bonds to generate isolated sites on the support [96].

#### 3.2.1. Mechanical Ball Milling

Mechanical ball milling is a scalable and solvent-free technique that utilizes shear and compressive forces to break metal-containing precursors and promote the formation of atomically dispersed active sites on support. Owing to its simplicity, environmental friendliness, and scalability, this method has emerged as a promising route for large-scale SAC production [97].

#### 3.2.2. Atom Trapping

**Figure 3:**
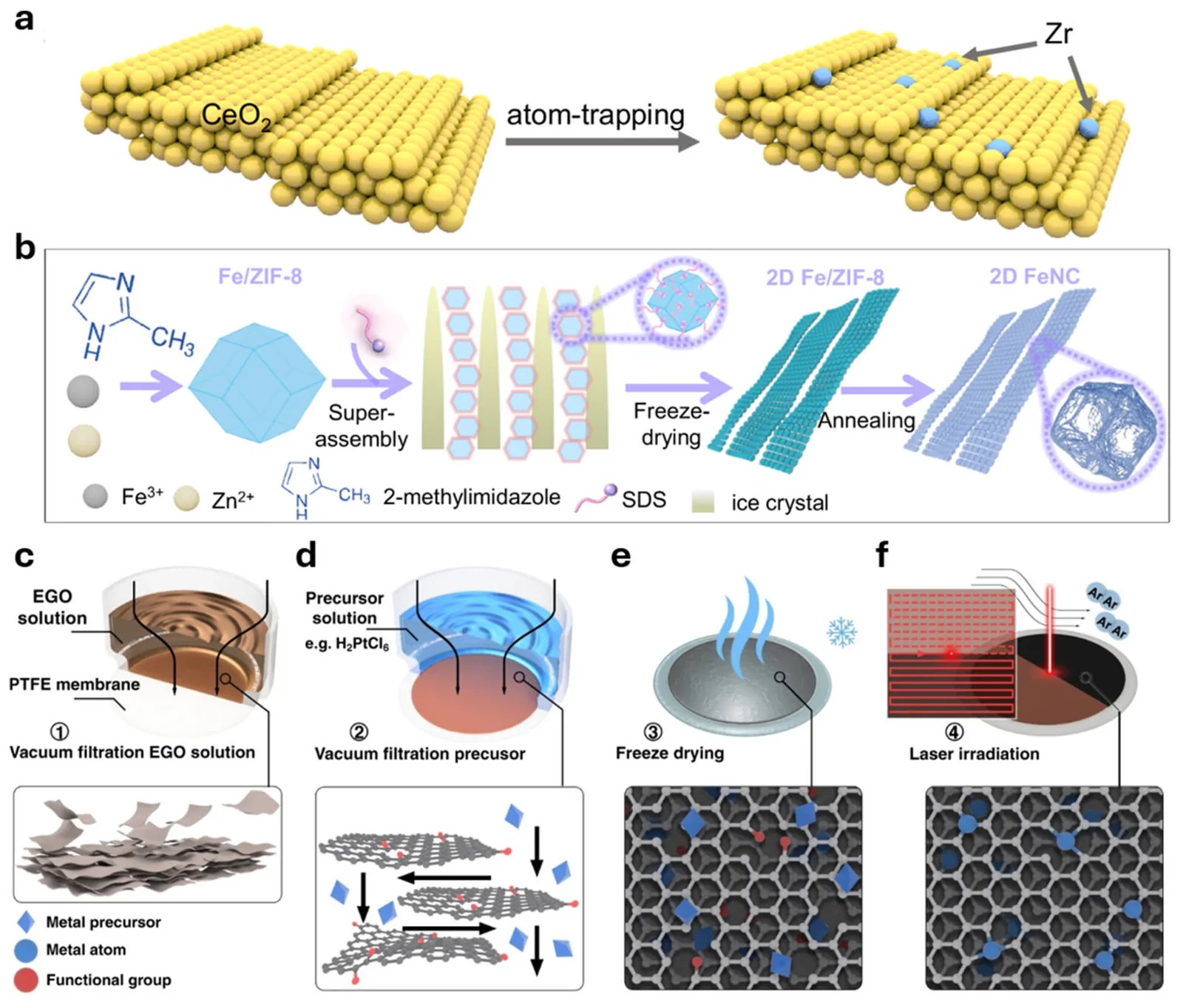
Representative top-down and confinement-assisted synthesis strategies for single-atom catalysts. (**a**) Schematic illustration of atom trapping for the preparation of Zr_1_–CeO_2_ through stabilization of isolated Zr atoms on a reducible oxide support. (**b**) Schematic illustration of the host–guest/freeze-casting-assisted synthesis of 2D Fe–N–C catalysts using Fe/ZIF-8 as the confinement matrix. (**c**–**f**) Laser-assisted synthesis of Pt single atoms on graphene, including EGO film preparation, Pt precursor incorporation, freeze-drying, and laser irradiation, to simultaneously induce graphitization and atomic dispersion of Pt species. Adapted from Refs. [98,99,101].

Atom trapping exploits surface defects, vacancies, or strong anchoring sites in reducible oxides to capture mobile metal atoms released during high-temperature treatment. As illustrated in Figure 3a, isolated Zr atoms can be stabilized on CeO_2_ through an atom-trapping process, resulting in the formation of Zr_1_–CeO_2_. This strategy demonstrates the ability of reducible oxide supports to immobilize individual atoms and prevent their aggregation, thereby producing stable atomically dispersed catalytic sites [98].

#### 3.2.3. Host–Guest Strategy

The host–guest strategy relies on the spatial confinement of metal precursors within porous host materials to suppress aggregation during subsequent thermal treatment. A representative example is shown in Figure 3b, where Fe/ZIF-8 serves as a host matrix for the confinement of Fe species. Combined with a surfactant-assisted freeze-casting process, this approach enables the formation of two-dimensional mesoporous Fe–N–C materials with highly accessible and atomically dispersed Fe active sites. The confinement effect provided by the host framework plays a critical role in preserving atomic dispersion during pyrolysis [99].

#### 3.2.4. Abrasion and Laser Planting

Abrasion utilizes direct mechanical contact between a bulk metal and a support to atomize metal species without significant material loss [100]. Laser planting, alternatively known as laser-assisted synthesis, employs laser irradiation to simultaneously generate anchoring defects and convert metal precursors into isolated active sites. As shown in Figure 3c–f, a Pt precursor is first incorporated into an electrochemical graphene oxide (EGO) film, followed by rapid freezing, freeze-drying, and laser irradiation. The laser treatment simultaneously induces graphitization of the support and conversion of the Pt precursor into atomically dispersed Pt sites, providing a rapid, scalable, and highly efficient route for SAC synthesis [101].

## 4. Support Engineering and Coordination Environments

Support materials are far more than passive scaffolds in single-atom catalysts (SACs); they play a central role in determining catalytic activity and stability. Their primary functions are to (i) anchor isolated metal atoms and prevent aggregation caused by their high surface free energy [102]; (ii) regulate the electronic structure and valence state of the active metal centers through strong metal–support interactions (MSIs), thereby optimizing the adsorption and desorption of reaction intermediates [103]; and (iii) actively participate in the catalytic process by facilitating charge transfer, mass transport, and intermediate stabilization [104].

### 4.1. Carbonaceous Substrates

Carbon-based materials, including graphene, carbon nanotubes (CNTs), graphitic carbon nitride (g-C_3_N_4_), and porous carbon, are among the most widely used SAC supports because of their high electrical conductivity, large specific surface area, excellent chemical stability, and relatively low cost [105].

Heteroatom Doping (N, S, P): Introducing heteroatoms into the carbon framework creates stable anchoring sites, typically in the form of M–Nx coordination, which effectively immobilize isolated metal atoms through strong coordination interactions [106].

Nitrogen (N) Doping: Nitrogen is the most widely employed dopant, providing pyridinic, pyrrolic, and graphitic N species that strongly coordinate metal atoms [107]. For example, Pt single atoms anchored on N-doped carbon (Pt_1_/N–C) exhibit a HER overpotential of only 19 mV at 10 mA cm^−2^, outperforming commercial Pt/C [108].

Sulfur (S) and Phosphorus (P) Doping: Sulfur and phosphorus modify the local electron density because of their larger atomic size and lower electronegativity than nitrogen, thereby tuning the electronic structure of the active metal center [109]. Accordingly, P-coordinated Ni–N_3_–P/CNFs have demonstrated excellent overall water-splitting performance with a low cell voltage of 1.67 V [110].

Vacancy Engineering: Carbon vacancies or nitrogen vacancies act as strong anchoring sites for isolated metal atoms, suppressing aggregation while optimizing intermediate adsorption. For instance, Pt–C_3_ sites on defective graphene exhibit approximately 18-fold higher HER activity than commercial Pt/C [111].

### 4.2. Transition Metal Compounds (TMCs)

Transition metal compounds (TMCs), including oxides, hydroxides, phosphides, and chalcogenides, provide abundant defects, vacancies, and unsaturated coordination sites that effectively stabilize isolated metal atoms while actively participating in water-splitting reactions.

Metal Oxides (TiO_2_, NiO, CeO_2_, and MnO_2_): Oxygen vacancies and surface hydroxyl groups serve as strong anchoring sites for isolated metal atoms [112]. For example, Ir single atoms supported on NiO, with loadings up to 18 wt%, deliver OER current densities approximately 46 times higher than IrO_2_ owing to strong covalent Ir–O interactions [76].

Layered Double Hydroxides (LDHs): LDHs, such as NiFe-LDH and CoFe-LDH, possess high surface areas, tunable compositions, and unique confinement effects that stabilize isolated active sites [113,114]. Ru single atoms anchored on NiFe-LDH optimize the adsorption energies of HER and OER intermediates, making them highly efficient bifunctional catalysts for overall water splitting [115].

Metal Phosphides and Chalcogenides (TMDs): These materials combine high electrical conductivity with favorable electronic structures similar to those of noble metals [116,117].

### 4.3. MXenes and Atomically Thin Materials (ATMs)

MXenes and other atomically thin materials (ATMs) have emerged as attractive SAC supports because of their metallic conductivity, hydrophilic surfaces, and abundant surface functional groups that strongly stabilize isolated metal atoms [118].

Pt on Mo_2_TiC_2_T_x_: Pt single atoms anchored at Mo vacancies (V_Mo_) in Mo_2_TiC_2_Tx MXene exhibit approximately 40 times higher HER mass activity than commercial Pt/C, highlighting the effectiveness of vacancy engineering in MXene-based SACs [119].

Strain Engineering: Tailoring the local strain through curvature modulation of nanoporous MoS_2_ enables precise regulation of the electronic structure of Ru single atoms, leading to excellent HER activity with an overpotential as low as 30 mV [120].

### 4.4. Coordination Environment Tuning

The catalytic performance of SACs can be systematically optimized by engineering the local coordination environment of the isolated metal atom. Unlike the mechanistic role of coordination discussed in Section 2.2.3, coordination environment tuning focuses on the deliberate modification of coordination geometry, coordination number, and ligand identity to regulate the electronic structure and catalytic properties of single-atom active sites.

Coordination Number (CN) Control: Reducing the coordination number from conventional M–N_4_ to lower-coordination configurations such as M–N_3_ or M–N_2_ creates more unsaturated and electronically active sites, improving intermediate adsorption and catalytic activity [121]. Numerous studies have demonstrated that lower-coordination SACs exhibit enhanced water electrolysis performance owing to the increased accessibility and intrinsic activity of the isolated metal centers.

Axial Coordination: Introducing axial ligands, including Cl, O, P, or S, provides an effective strategy for fine-tuning the electronic structure of the active metal center without altering the primary coordination framework [122]. For example, Cl-coordinated Pt/LDH optimizes the adsorption of both H* and OH* intermediates, thereby improving the kinetics of both HER and OER and enhancing overall water-splitting performance [123].

Overall, coordination environment engineering provides a versatile approach for tailoring the intrinsic catalytic properties of SACs by controlling the local electronic structure, intermediate adsorption behavior, and reaction kinetics. Together with support engineering and defect modulation, it represents one of the most powerful design strategies for developing highly active and durable single-atom electrocatalysts for water electrolysis.

### 4.5. Waste-Derived Supports: A Sustainable Dimension

Developing SAC supports from renewable biomass and industrial waste has emerged as a sustainable strategy that integrates catalyst design with circular economy principles [124].

Waste Yeast Biomass: Carbonized waste yeast has been employed as a low-cost support for Ru single atoms, producing a catalyst with a high exchange current density (j_0_) of 5.84 mA cm^−2^, outperforming commercial Ru/C for HER [36].

Radioactive Wastewater: Uranium single atoms recovered from radioactive wastewater have been successfully anchored onto MoS_2_ nanosheets with a loading of 5.2%, delivering a HER overpotential of only 72 mV while simultaneously demonstrating a sustainable route for waste resource utilization [125].

Although extensive progress has been achieved in support engineering for single-atom catalysts, no universal support material has yet demonstrated superiority across all performance metrics. Carbonaceous substrates remain the most extensively investigated supports because of their excellent electrical conductivity, large surface area, and structural tunability. In particular, heteroatom doping and vacancy engineering provide effective anchoring sites that suppress metal aggregation while simultaneously modulating the electronic structure of isolated metal centers. However, the relatively inert nature of carbon supports limits their direct participation in catalytic reactions, and the density and distribution of defects are often difficult to control reproducibly, leading to variations in catalytic performance among different studies.

In contrast, transition metal compounds (TMCs), including oxides, hydroxides, phosphides, and chalcogenides, provide stronger metal–support interactions and actively participate in the catalytic process through electronic coupling with the isolated metal atoms. Layered double hydroxides and transition metal chalcogenides, for example, have consistently demonstrated superior bifunctional HER/OER performance because both the support and the anchored single atoms contribute to the reaction. Nevertheless, these materials frequently exhibit lower intrinsic electrical conductivity than carbon-based supports, requiring additional conductivity-enhancing strategies. Furthermore, structural reconstruction of TMC supports under electrochemical operating conditions, particularly during oxygen evolution, complicates identification of the true catalytically active species and remains an important unresolved issue.

MXenes and other atomically thin materials represent an emerging class of supports that combine metallic conductivity with abundant surface functional groups capable of strongly stabilizing isolated metal atoms. Compared with conventional carbon supports, MXenes generally facilitate faster charge transport and stronger electronic interactions with single-atom centers. However, their practical implementation is still hindered by challenges associated with large-scale synthesis, susceptibility to oxidation during long-term operation, and the limited understanding of the stability of surface terminations under electrochemical conditions.

Coordination environment engineering has emerged as one of the most effective approaches for optimizing intrinsic catalytic activity because subtle changes in coordination number, ligand identity, or local symmetry can significantly alter the electronic structure and adsorption energies of reaction intermediates. However, accurately identifying the real coordination environment under operating conditions remains challenging because electrochemical reconstruction, ligand exchange, and dynamic coordination changes may occur during HER and OER. Consequently, correlations between the initially synthesized structure and the actual active site are not always straightforward, highlighting the importance of operando spectroscopic characterization and theoretical modeling.

Although waste-derived supports offer an environmentally sustainable and economically attractive alternative to conventional materials, their structural heterogeneity, compositional variability, and limited reproducibility currently restrict large-scale application. Future research should therefore focus on balancing catalytic activity, structural stability, conductivity, scalability, and sustainability, while establishing standardized protocols for evaluating the influence of support properties on intrinsic single-atom catalytic activity. Such systematic comparisons will be essential for developing universal design principles for next-generation SAC supports.

## 5. Structural Characterization and Identification of SACs

Unraveling the structure–activity relationship in single-atom catalysts (SACs) requires characterization techniques with atomic resolution. Because isolated metal atoms lack long-range periodicity and are often present in low concentrations, traditional tools like X-ray Diffraction (XRD) and conventional Transmission Electron Microscopy (TEM) are insufficient to confirm their existence. Advanced microscopy and spectroscopy are necessary to identify the geometric configuration, oxidation state, and local coordination environment of the active sites.

### 5.1. Aberration-Corrected Scanning Transmission Electron Microscopy (AC-STEM)

**Figure 4:**
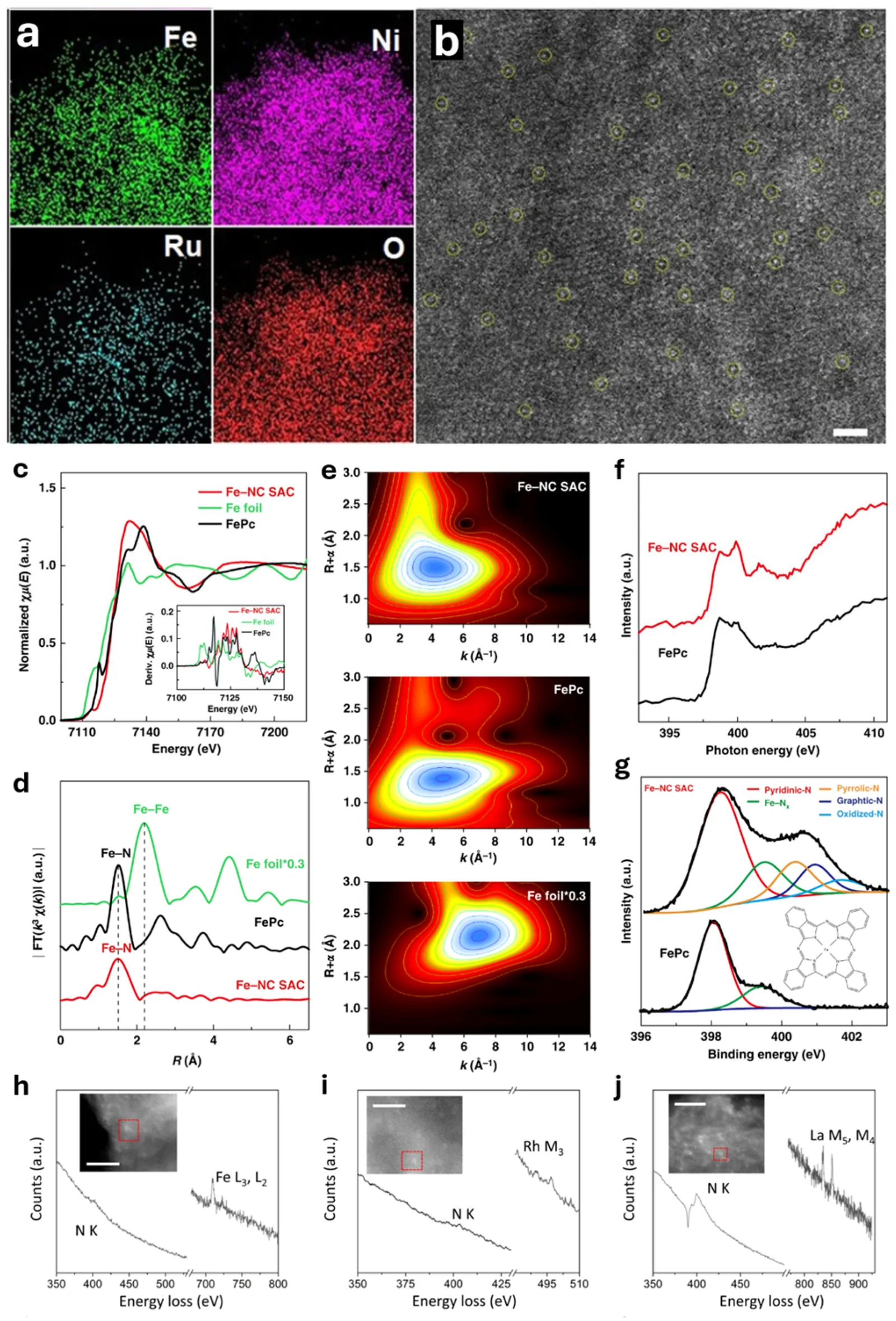
Advanced characterization of single-atom catalysts. (**a**) Elemental mapping and (**b**) aberration-corrected HAADF-STEM image of Ru_1_/D-NiFe LDH, showing the homogeneous distribution of elements and isolated Ru atoms (yellow circles) with atomic dispersion on the LDH support (scale bar 1 nm). Adapted from ref. [129]. (**c**) Fe K-edge XANES spectra (inset: first-derivative curves), (**d**) Fourier-transformed Fe K-edge EXAFS spectra, and (**e**) wavelet-transform EXAFS analysis of Fe–NC SAC and reference samples (FePc and Fe foil), confirming the oxidation state and Fe–Nx coordination environment of isolated Fe sites. (**f**) N K-edge NEXAFS spectra and (**g**) deconvoluted N 1s XPS spectra of Fe–NC SAC and FePc, providing further evidence for Fe–N coordination and electronic structure modulation. Adapted from ref. [135]. (**h**–**j**) Atomic-resolution EELS spectra of Fe-, Rh-, and La-based M–N–C catalysts, showing the N K-edge and corresponding metal-edge signals, which verify nitrogen coordination around isolated M–Nx active sites. Adapted from ref. [140].

AC-STEM, particularly in High-Angle Annular Dark-Field (HAADF) mode, is the primary technique for directly visualizing isolated metal atoms in SACs [126]. HAADF-STEM imaging relies on incoherent elastic scattering, where the signal intensity is approximately proportional (Z^1.7−2^), where *Z* is the atomic number [127]. Consequently, heavy transition metal atoms (e.g., Pt, Ir, Ru) appear as isolated bright spots against the darker background of low-Z supports like carbon, nitrogen-doped carbon, and layered hydroxides [128]. A representative example is Ru_1_/D-NiFe LDH, where atomically dispersed Ru species are clearly observed as isolated bright spots uniformly distributed on the support surface without detectable nanoparticles or clusters (Figure 4b) [129]. The corresponding elemental mapping further demonstrates the homogeneous spatial distribution of Ru together with the host elements, confirming successful atomic dispersion throughout the catalyst matrix (Figure 4a) [129].

The aberration-corrected STEM technique provides atomic-scale spatial resolution (~0.1–0.2 nm), enabling direct verification of single-atom configurations. Such capability enables the identification of isolated atoms, their spatial distribution, and, in some cases, their incorporation into specific lattice positions of the host material [76]. Therefore, AC-STEM is widely regarded as a primary characterization technique for verifying the atomic dispersion of SACs.

### 5.2. X-Ray Absorption Spectroscopy (XAS)

X-ray Absorption Spectroscopy (XAS) provides detailed information regarding the local coordination environment and electronic structure of atomically dispersed metal centers, making it one of the most important techniques for SAC characterization [130].

X-ray Absorption Near-Edge Structure (XANES) analysis is commonly used to determine the oxidation state and coordination geometry of metal atoms [131]. The near-edge absorption energy and the intensity of the “white line” peak reflect the density of unoccupied states (e.g., metal 5d orbitals) [132,133,134]. As shown in the Fe K-edge XANES spectra of Fe–NC SAC (Figure 4c), the absorption-edge position lies between those of metallic Fe and Fe phthalocyanine references, indicating an intermediate oxidation state of the Fe centers and strong interaction with the surrounding coordination environment [135].

Extended X-ray Absorption Fine Structure (EXAFS) analysis provides quantitative information regarding coordination numbers and bond distances around the active metal centers [133]. A key feature of SACs is the absence of metal–metal coordination signals in the Fourier-transformed EXAFS spectra. In the Fe–NC catalyst, the dominant scattering contribution originates from Fe–N coordination, while no Fe–Fe coordination peak is observed (Figure 4c), confirming the atomic dispersion of Fe species [135].

To further distinguish different scattering paths, wavelet-transform EXAFS (WT-EXAFS) combines both radial and momentum-space information. The WT contour plot of Fe–NC SACs displays a characteristic feature corresponding to Fe–N coordination and lacks signatures associated with Fe–Fe scattering (Figure 4e), providing additional evidence for the formation of isolated Fe–Nx active sites [135].

### 5.3. X-Ray Photoelectron Spectroscopy (XPS)

X-ray Photoelectron Spectroscopy (XPS) is a surface-sensitive technique (depth < 2 nm) used to evaluate the valence state and elemental composition of the catalyst surface [136]. Binding-energy shifts provide valuable information regarding oxidation-state variations and electronic interactions between the metal centers and support [137]. For the Fe–NC catalyst, N K-edge spectroscopy and deconvoluted N 1s XPS spectra reveal the presence of different nitrogen species and their interaction with the atomically dispersed Fe centers (Figure 4f,g) [135]. The formation of Fe–N_x_ coordination environments is evidenced by the characteristic nitrogen-related features, while changes in spectral intensity indicate electronic coupling between Fe atoms and the nitrogen-doped carbon framework. Such information is essential for understanding charge redistribution and active-site formation in SACs.

### 5.4. Electron Energy Loss Spectroscopy (EELS) and EDS Mapping

Electron Energy Loss Spectroscopy (EELS), when integrated with STEM, enables atomic-scale analysis of the local chemical and electronic environment, particularly for light elements such as carbon, nitrogen, and oxygen [138]. Because of its high spatial resolution, EELS can directly probe the coordination environment surrounding individual metal atoms [139]. As shown in Figure 4h–j, atomic-resolution EELS spectra collected from individual Fe, Rh, and La single-atom sites exhibit both the characteristic metal-edge signals and the N K-edge response [140]. The simultaneous detection of these features provides direct evidence that the isolated metal atoms are coordinated by nitrogen atoms, confirming the formation of M–Nx active sites at the atomic scale.

Complementary to EELS, Energy-Dispersive X-ray Spectroscopy (EDS) mapping is frequently employed to visualize the spatial distribution of elements within SACs [141]. Uniform elemental distributions obtained from EDS mapping provide additional evidence for the homogeneous dispersion of active species and help exclude the presence of aggregated metal clusters [133].

### 5.5. In-Situ and Operando Characterization

In-situ and operando characterization techniques provide direct insight into the dynamic evolution of active sites, electronic structures, and reaction intermediates under realistic operating conditions, enabling a deeper understanding of catalytic mechanisms during water splitting [142].

**Figure 5:**
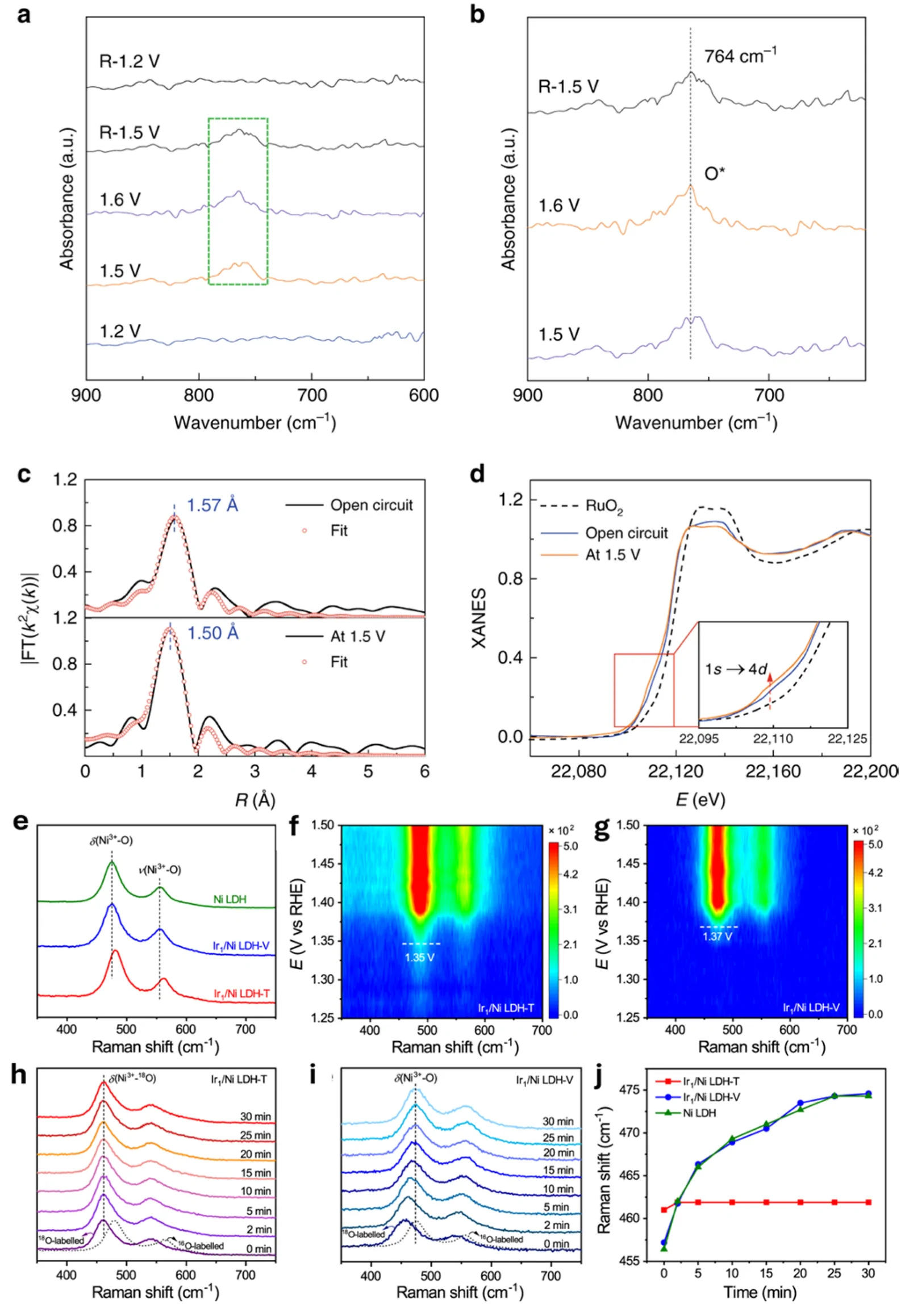
In-situ and operando characterization of single-atom catalysts during OER. (**a**,**b**) Operando SR-FTIR spectra of Ru–N–C, showing potential-dependent evolution of surface intermediates. (**c**,**d**) Operando EXAFS and XANES spectra of Ru–N–C at different applied potentials, revealing changes in the local coordination environment and electronic structure of Ru sites. Adapted from Ref. [144]. (**e**–**j**) In-situ Raman spectra of Ir1/Ni LDH catalysts, including isotope-exchange measurements, providing insight into catalyst reconstruction, lattice oxygen participation, and active-site evolution during OER. Adapted from Ref. [146].

Operando XAS: X-ray absorption spectroscopy performed under working conditions can monitor changes in oxidation state and local coordination environment during electrocatalysis [143]. As illustrated in Figure 5c,d, operando XANES and EXAFS analyses of Ru–N–C during OER reveal the evolution of the Ru electronic structure and local coordination environment with applied potential, providing direct evidence of the structural stability and catalytic behavior of the isolated Ru sites [144].

Operando FTIR Spectroscopy: Operando FTIR enables the detection of surface-adsorbed reaction intermediates and changes in chemical bonding during catalysis. The operando SR-FTIR spectra of Ru–N–C (Figure 5a,b) show potential-dependent infrared features associated with reaction intermediates formed during OER, demonstrating the capability of FTIR spectroscopy to track reaction pathways in real time [144].

In-situ Raman Spectroscopy: In-situ Raman spectroscopy is widely used to monitor catalyst reconstruction, phase evolution, and intermediate formation under reaction conditions [145]. As shown in Figure 5e–j, in-situ Raman measurements of Ir1/Ni LDH catalysts reveal potential-dependent structural transformations and oxygen-isotope exchange behavior, providing insight into the participation of lattice oxygen and the evolution of active catalytic sites during OER [146].

## 6. Classification and Applications of Transition Metal Single-Atom Catalysts (TMSACs)

**Figure 6:**
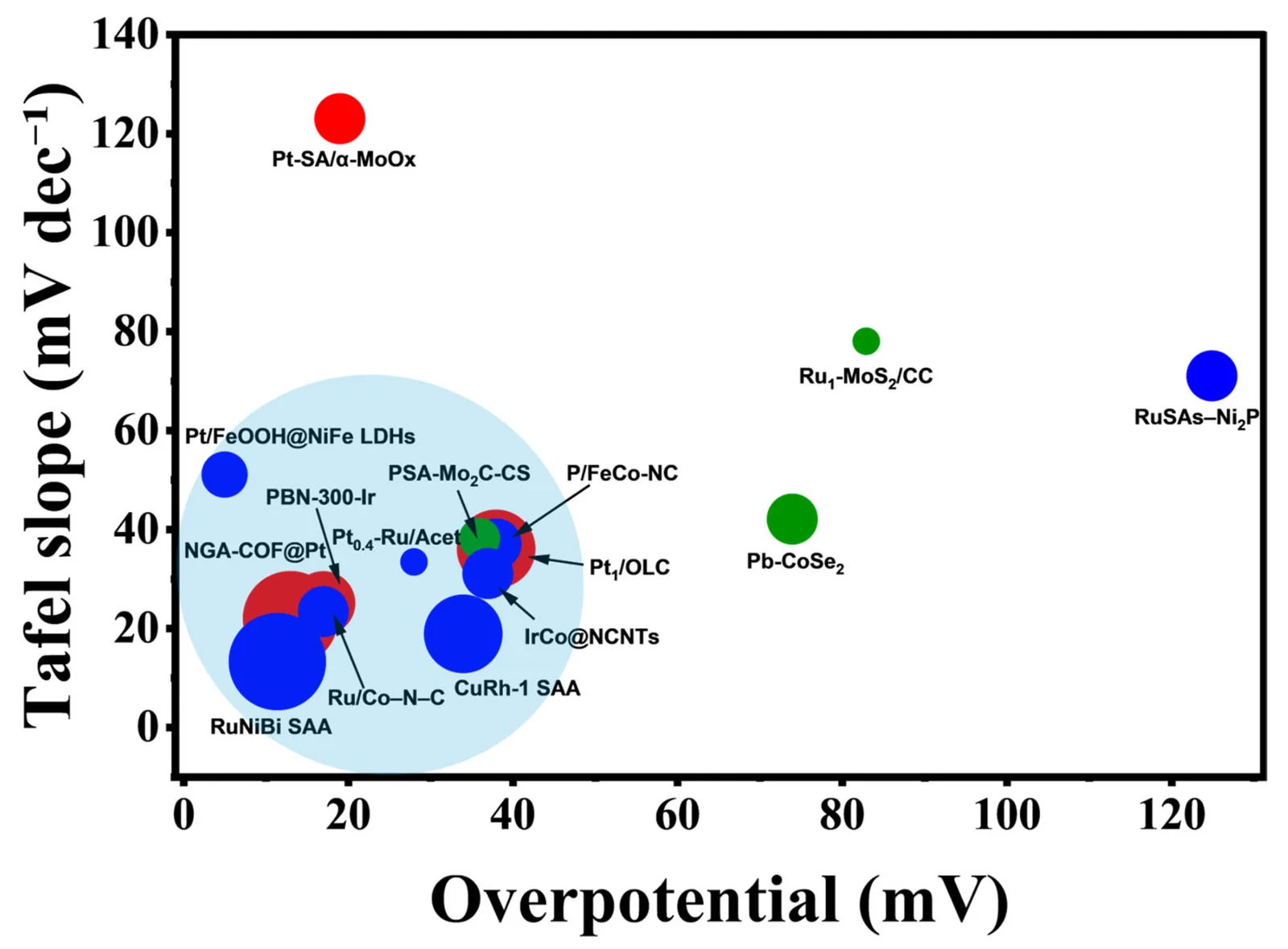
Bubble chart comparing the electrocatalytic hydrogen evolution reaction (HER) performance of single-atom catalysts (SACs). The *x*-axis represents the overpotential required to achieve 10 mA cm^−2^ (η_10_), while the *y*-axis represents the corresponding Tafel slope. Bubble size is proportional to the log_10_ of the reported stability (h), reflecting catalyst durability, and bubble color denotes catalyst class: red = noble-metal SACs, green = non-noble-metal SACs, and blue = dual/bimetallic catalysts.

**Figure 7:**
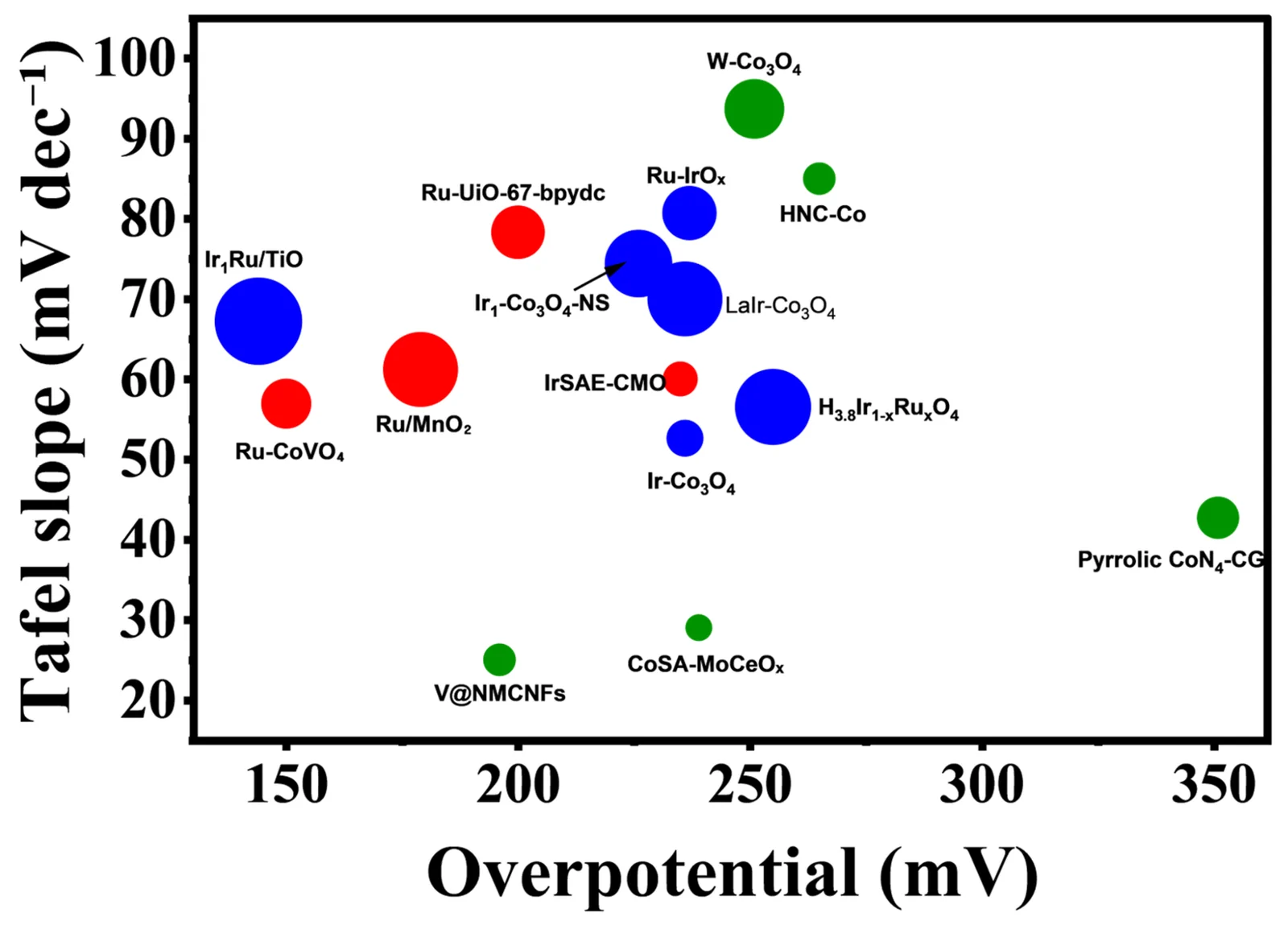
Bubble chart comparing the electrocatalytic oxygen evolution reaction (OER) performance of representative single-atom catalysts (SACs). The *x*-axis represents the overpotential required to achieve 10 mA cm^−2^ (η_10_), while the *y*-axis represents the corresponding Tafel slope. Bubble size is proportional to the logarithm of the reported stability (h), reflecting catalyst durability, and bubble color denotes catalyst class: red = noble-metal SACs, green = non-noble-metal SACs, and blue = dual/bimetallic catalysts.

Transition metal single-atom catalysts (TMSACs) can be broadly classified into noble-metal SACs, non-noble-metal SACs, and bimetallic or dual-atom catalysts (DACs) [147]. Their electrocatalytic performance is commonly evaluated using overpotential, Tafel slope, turnover frequency (TOF), mass activity, and long-term stability. Owing to their atomically dispersed active sites and tunable coordination environments, TMSACs have demonstrated remarkable activity toward both the hydrogen evolution reaction (HER) and oxygen evolution reaction (OER) [148]. However, the catalytic behavior of different SAC classes involves important trade-offs among intrinsic activity, stability, cost, and scalability, which must be carefully considered for practical hydrogen production. A quantitative comparison of representative HER and OER single-atom catalysts is summarized in Table 1 and Table 2, while their overall performance trends in terms of overpotential, Tafel slope, stability, and catalyst classification are visualized in Figure 6 and Figure 7.

### 6.1. Noble Metal SACs

Noble-metal SACs, including Pt, Ru, Ir, Rh, Pd, and Au, remain the benchmark electrocatalysts because of their intrinsically favorable adsorption energies for HER and OER intermediates. Among these systems, Pt-based SACs have demonstrated exceptional HER performance with significantly enhanced mass activity compared with commercial Pt/C by maximizing the utilization of individual metal atoms [149]. Ru-based SACs have emerged as attractive alternatives to Pt owing to their excellent HER performance and promising bifunctional activity for overall water splitting [150]. Similarly, Ir-based SACs remain among the most effective catalysts for acidic OER owing to their excellent intrinsic activity and corrosion resistance under highly oxidative conditions [151].

The high activity of noble-metal SACs originates from precise regulation of the metal electronic structure through coordination engineering and strong metal–support interactions, which optimize the adsorption energies of key reaction intermediates. However, despite the reduction in noble-metal consumption achieved through atomic dispersion, their large-scale application remains restricted by the high cost, limited availability, and potential dissolution of noble metals under harsh electrochemical environments. Therefore, noble-metal SACs are mainly considered benchmark systems for understanding catalytic mechanisms and establishing activity descriptors rather than ideal candidates for large-scale hydrogen production.

### 6.2. Non-Noble Metal SACs

To improve economic viability, increasing attention has been directed toward SACs based on earth-abundant transition metals, including Fe, Co, Ni, Mo, W, V, and Mn [152]. Although these catalysts generally exhibit lower intrinsic activity compared with noble-metal systems, their electronic structures can be effectively optimized through coordination engineering, heteroatom doping, defect modulation, and metal–support interactions.

Ni- and Co-based SACs have shown promising bifunctional HER/OER activity, particularly in alkaline electrolytes, where their oxophilic nature facilitates water activation and oxygen intermediate conversion. Fe-based SACs are widely investigated for OER because of their favorable electronic structures and ability to regulate oxygen intermediate adsorption. Meanwhile, Mo- and W-based SACs demonstrate excellent HER activity by providing suitable hydrogen adsorption characteristics, whereas V- and Mn-based SACs have shown potential for OER through modified oxygen intermediate binding [147]. Compared with noble-metal SACs, non-noble systems offer advantages in cost, abundance, and scalability; however, their practical performance is still limited by slower reaction kinetics, lower intrinsic conductivity, and reduced stability under aggressive operating conditions. In particular, the identification and preservation of the true active sites during long-term electrolysis remain challenging because reconstruction, oxidation state changes, and dynamic coordination rearrangements may occur under operating conditions. Recent studies exploring unconventional metal centers, such as uranium recovered from wastewater, further demonstrate the expanding design space of sustainable SACs but also highlight the need for improved stability and reproducibility.

### 6.3. Bimetallic and Dual-Atom Catalysts

Bimetallic and dual-atom catalysts (DACs) represent an emerging extension of SACs, where two neighboring metal atoms create synergistic active sites that are difficult to achieve with isolated single atoms [153]. Compared with conventional SACs, DACs provide additional flexibility for controlling electronic structures and reaction pathways through direct metal–metal interactions, charge redistribution, and cooperative adsorption of reaction intermediates.

The enhanced catalytic activity of DACs originates from the complementary functions of neighboring metal centers. During HER, one metal site can facilitate water dissociation or hydrogen adsorption, while the adjacent atom can promote hydrogen coupling and release. For OER, dual-metal centers can optimize the adsorption energies of OH*, O*, and OOH* intermediates and facilitate O–O bond formation by providing adjacent reaction sites. Consequently, DACs can partially overcome the adsorption-energy scaling relationships that limit conventional single-site catalysts.

DACs can be divided into homonuclear and heteronuclear systems. Homonuclear DACs, such as Fe–Fe, Co–Co, and Pt–Pt configurations, mainly rely on cooperative interactions between equivalent metal centers. In contrast, heteronuclear DACs, including Pt–Ru, Ni–Co, Fe–Ni, Ir–Mo, W–Mo, and Fe–Mo systems, provide greater tunability because two different metals can perform complementary catalytic functions [154,155]. For example, one metal center may optimize adsorption of a specific intermediate, whereas the second metal modifies the electronic environment or facilitates subsequent reaction steps, making heteronuclear DACs particularly attractive for bifunctional water electrolysis.

Despite these advantages, DACs face several challenges before practical implementation. The controlled synthesis of two neighboring metal atoms with precise atomic separation, coordination environment, and uniform distribution remains significantly more difficult than preparing SACs. Increasing metal loading may induce migration and aggregation, resulting in nanoparticles rather than true dual-atom sites. Moreover, distinguishing DACs from spatially separated single atoms requires advanced characterization approaches, including aberration-corrected HAADF-STEM, X-ray absorption spectroscopy (XAS), and theoretical calculations. The possibility of structural reconstruction during HER and OER operation further complicates identification of the real active structure.

**Table 1:** Representative transition-metal single-atom and dual-atom electrocatalysts reported for the hydrogen evolution reaction (HER) together with their electrochemical performance.

Catalyst System	Electrolyte	Overpotential at 10 mA cm^−2^, η_10_ (mV)	Tafel Slope (mV dec^−1^)	Durability Assessment	Reference
Noble-Metal Single-Atom Catalysts (SACs)					
Pt_1_/OLC	N_2_-saturated 0.5 M H_2_SO_4_	38	36	6000 cycles; 100 h at 10 mA cm^−2^	[156]
Pt SA/WO_3−x_	H_2_-saturated 0.5 M H_2_SO_4_	47	45	10 h at −40 mV_RHE_	[157]
Pt-SA/α-MoO_x_	H_2_-saturated 0.5 M H_2_SO_4_	19	123	5000 cycles; 20 h at 10 mA cm^−2^	[158]
PBN-300-Ir	0.5 M H_2_SO_4_	17	25	>42 h at 10 mA cm^−2^	[159]
RuSA@2H-MoS_2_	0.5 M H_2_SO_4_	167	77.5	3000 cycles	[160]
Ru_1_–1T-MoS_2_/CC	N_2_-saturated 0.5 M H_2_SO_4_	83	78	—	[161]
PtO_4_@MX	0.5 M H_2_SO_4_	33.1	27.2	80 h at 20 mA cm^−2^	[162]
NGA-COF@Pt	0.5 M H_2_SO_4_	13	21.88	260 h at 100 mA cm^−2^	[163]
PANI@Pt/S-TiN NTs/Ti	N_2_-saturated 0.5 M H_2_SO_4_	12	26.9	25 h at 10 mA cm^−2^	[164]
**Non-Noble-Metal Single-Atom Catalysts (SACs)**					
P/FeCo-NC	Ar-saturated 0.5 M H_2_SO_4_	38	37	2000 cycles	[165]
Pb-CoSe_2_-DETA	0.5 M H_2_SO_4_	74	42	1000 cycles; 20 h at 10 mA cm^−2^	[166]
**Dual-Atom, Bimetallic, and Single-Atom Alloy Catalysts**					
IrSA@Fe@NCNT	0.5 M H_2_SO_4_	26	31.8	12 h at 10 mA cm^−2^	[167]
RuSAs–Ni_2_P	0.5 M H_2_SO_4_	125	71	2000 cycles	[168]
Ru/Co–N–C	0.5 M H_2_SO_4_	17	23.3	>20 h at 100 mA cm^−2^	[130]
Co/Pd_m_-4	N_2_-saturated 0.5 M H_2_SO_4_	24.7	8.2	5000 cycles; 42 h at 50 mA cm^−2^	[169]
Pt_1_+Cs-NPC	0.5 M H_2_SO_4_	24	12.5	50 h at 20 mA cm^−2^	[170]
IrCo@NCNTs	0.5 M H_2_SO_4_	37	31	2000 cycles; 20 h at 10 mA cm^−2^	[171]
Ru–O–MoS_2_	0.5 M H_2_SO_4_	16	35	40 h at 10 mA cm^−2^	[172]
Pt/FeOOH@NiFe LDHs	N_2_-saturated 0.5 M H_2_SO_4_	5	51	15 h at 10 mA cm^−2^	[173]
CuRh-1 SAA	0.5 M H_2_SO_4_	34	18.8	100 h at 10 mA cm^−2^; 25 h at 100 mA cm^−2^	[174]
RuNiBi SAA	Ar-saturated 0.5 M H_2_SO_4_	11.4	13.16	300 h at 10 mA cm^−2^; 500 h at 1 mA cm^−2^ (PEMWE)	[175]
Pt_0_._47_–Ru/Acet	0.5 M H_2_SO_4_	28	33.34	—	[176]

**Table 2:** Representative transition-metal single-atom and dual-atom electrocatalysts reported for the oxygen evolution reaction (OER) together with their electrochemical performance.

Catalyst System	Electrolyte	η_10_ (mV)	Tafel Slope (mV dec^−1^)	Durability Assessment	Ref.
Noble-Metal Single-Atom Catalysts (SACs)					
IrSAE-CMO	Ar-saturated 0.5 M H_2_SO_4_	235	60	25 h at 10 mA cm^−2^	[177]
Ir-NiCo_2_O_4_ NSs	H_2_-saturated 0.5 M H_2_SO_4_	240	60	70 h at 10 mA cm^−2^	[178]
Pt_1_–C_2_N_2_	0.5 M H_2_SO_4_	232	87	12 h at 1.462 VRHE	[179]
Ir_1_-Co_3_O_4_-NS	0.5 M H_2_SO_4_	226	74.4	500 h at 10 mA cm^−2^	[180]
Ru-UiO-67-bpydc	0.5 M H_2_SO_4_	200	78.3	1000 cycles; 140 h at 50 mA cm^−2^	[181]
ZnSA-RuO_2_	0.1 M HClO_4_	210	56	1000 cycles; 43 h at 10 mA cm^−2^	[182]
IrVI-ado	—	—	—	2700 h at 1.8 A cm^−2^ (PEMWE)	[183]
Ir_1_-PI@CP	0.5 M H_2_SO_4_	254	51	7000 cycles; 360 h at 10 mA cm^−2^	[184]
ICM-activated	0.5 M H_2_SO_4_	232	54.5	250 h at 10 mA cm^−2^; 400 h at 1 mA cm^−2^ (PEMWE)	[185]
Ru/MnO_2_	0.5 M H_2_SO_4_	179	61.2	1000 h at 100 mA cm^−2^; 500 h at 500 mA cm^−2^ (PEMWE)	[186]
COF-205-Ru	0.5 M H_2_SO_4_	210	70	280 h	[187]
Ta-RuO_2_	0.1 M HClO_4_	201	55	280 h	[188]
Ru-SA/Ti_3_C_2_T_x_	0.1 M HClO_4_	290	37.9	32 h	[189]
**Non-noble metal SACs**					
V@NMCNFs	0.5 M H_2_SO_4_	196	25.0	20 h at 20/40/60 mA cm^−2^	[190]
CoSA–MoCeO_x_	0.5 M H_2_SO_4_	239	29.0	12 h at 10 mA cm^−2^	[191]
W–Co_3_O_4_	0.5 M H_2_SO_4_	251	93.7	250 h; 240 h at 1 A cm^−2^	[192]
Pyrrolic CoN_4_–CG	0.5 M H_2_SO_4_	351	42.7	50 h at 10 mA cm^−2^	[193]
HNC–Co	0.5 M H_2_SO_4_	265	85.0	20 h at 40 mA cm^−2^	[194]
**Dual-atom/Bimetallic catalysts**					
Ru–IrO_x_	0.5 M H_2_SO_4_	237	80.7	150 h at 100 mA cm^−2^	[195]
H_3_._8_Ir_1−x_Ru_x_O_4_	0.5 M H_2_SO_4_	255	56.5	1100 h at 10 mA cm^−2^; 1280 h at 2 A cm^−2^ (PEMWE)	[75]
Ir–RuO_2_	0.5 M H_2_SO_4_	167	48.2	200,000 cycles; >1000 h at 10 mA cm^−2^	[196]
LaIr–Co_3_O_4_	0.5 M H_2_SO_4_	236	70.0	1000 h at 10 mA cm^−2^; 1000 h at 1 A cm^−2^ (PEMWE)	[197]
Ru–Co_2_MnO_4_._5_	0.5 M H_2_SO_4_	176	29.0	600 h at 10 mA cm^−2^; 100 h at 1 A cm^−2^ (PEMWE)	[198]
Ir_1_Ru/TiO_2_	0.5 M H_2_SO_4_	144	67.2	3200 h at 10 mA cm^−2^; 2000 h at 2 A cm^−2^ (PEMWE)	[199]
Ru–CovO_4_	0.5 M H_2_SO_4_	150	56.9	4000 cycles; 100 h at 10 mA cm^−2^	[200]
Mn(SAs)–Ru/RuO_2_	0.5 M H_2_SO_4_	158	86.2	150 h at 10 mA cm^−2^	[201]
Ru(anc)–Co_3_O_4_	0.5 M H_2_SO_4_	198.5	49.2	150 h	[202]
Ru(SA)–NiOMS	0.5 M H_2_SO_4_	160	32.8	300 h; 100 h at 100 mA cm^−2^	[203]
Ru_1_–Pt_3_Cu	0.1 M HClO_4_	220	—	28 h	[204]

### 6.4. Critical Comparison and Benchmarking Considerations

Although numerous TMSAC systems demonstrate exceptional electrocatalytic performance, direct comparison among different catalyst classes requires careful interpretation. Reported activity values are often obtained under different electrolyte compositions, catalyst loadings, substrate configurations, current densities, and normalization methods, which can lead to misleading comparisons. For example, a lower overpotential does not always indicate higher intrinsic activity if differences in active-site density, mass loading, or surface accessibility are not considered.

Noble-metal SACs generally provide the highest intrinsic activity but suffer from cost and resource limitations. Non-noble SACs offer improved economic feasibility but often require further optimization of electronic structure and stability. DACs provide a promising intermediate strategy by combining the atomic efficiency of SACs with additional cooperative interactions; however, their structural complexity and uncertain active-site evolution remain important challenges.

Therefore, future progress requires standardized evaluation protocols involving consistent measurements of overpotential, Tafel slope, TOF, mass activity, Faradaic efficiency, and long-term durability. Integration of operando characterization with theoretical modeling will also be essential for establishing reliable structure–activity relationships and accelerating the development of practical SAC and DAC electrocatalysts for large-scale water electrolysis.

## 7. Challenges and Future Perspectives in Single-Atom Electrocatalysis

Despite the remarkable progress of single-atom catalysts (SACs) for water splitting, several challenges still hinder their practical deployment. Future research should focus not only on maximizing catalytic activity but also on improving scalability, stability, mechanistic understanding, and economic viability.

### 7.1. Scalable Synthesis and High Metal Loading

Most SACs are still produced at laboratory scale using synthesis routes that are difficult to translate into industrial manufacturing. Developing scalable and cost-effective methods, such as ball milling, Joule heating, microwave-assisted synthesis, and pulsed discharge techniques, is therefore essential [205]. At the same time, increasing metal loading while maintaining atomic dispersion remains a major challenge, as isolated atoms tend to aggregate because of their high surface free energy. Advanced supports with abundant anchoring sites, including defect-rich carbons and metal oxides, have enabled significantly higher loadings, but preserving atomic dispersion under operating conditions remains critical.

### 7.2. Stability Under Practical Operating Conditions

Long-term durability is one of the most important requirements for industrial water electrolysis. During extended operation, isolated metal atoms may migrate, aggregate, or leach into the electrolyte, leading to performance degradation. These issues are particularly severe under harsh OER conditions. Moreover, the long-term structural stability of SACs remains a subject of ongoing debate. Although many studies attribute performance degradation to atom migration and aggregation, others have reported dynamic catalyst reconstruction, in which the initially synthesized single-atom sites evolve into new coordination environments or sub-nanometer clusters under operating conditions. Consequently, the catalytically active species responsible for the observed activity may differ from those identified before electrochemical testing. Distinguishing reversible structural evolution from irreversible catalyst degradation therefore remains an important challenge for the rational design of durable SACs. Strengthening metal–support interactions through defect engineering, heteroatom doping, and coordination environment optimization will be crucial for improving catalyst stability and maintaining active-site integrity over prolonged operation [150].

### 7.3. Mechanistic Understanding Through Operando Studies

Although SACs possess well-defined active sites, the true catalytic species under operating conditions often remain unclear. Surface reconstruction, changes in oxidation state, and dynamic coordination environments can occur during HER and OER, making the identification of active sites challenging [155]. Advanced operando characterization techniques, including XAS, Raman, FTIR, and environmental TEM, combined with realistic theoretical modeling, are needed to reveal reaction pathways and structure–activity relationships under practical electrochemical conditions. Another unresolved issue concerns the identification of the true active sites responsible for catalytic activity. Although aberration-corrected HAADF-STEM and X-ray absorption spectroscopy provide valuable structural information, these techniques often characterize catalysts either before or after electrochemical operation and may not fully capture the dynamic evolution of isolated metal centers under realistic reaction conditions. Furthermore, discrepancies between experimental observations and theoretical predictions have led to ongoing debates regarding whether the initially dispersed single atoms, reconstructed coordination environments, or transient multinuclear species constitute the actual catalytic centers. Addressing these uncertainties will require the combined use of advanced operando characterization techniques and multiscale theoretical modeling.

### 7.4. Data-Driven Catalyst Design and Standardization

The enormous chemical space of possible metal–support combinations makes conventional trial-and-error catalyst discovery both time-consuming and resource-intensive. Integrating machine learning with high-throughput computational screening and density functional theory offers a promising strategy for accelerating the rational design of next-generation SACs [206]. Equally important is the establishment of standardized electrochemical testing and reporting protocols. Direct comparison among reported SACs is often complicated by differences in catalyst loading, electrolyte composition, electrode preparation, current density, iR compensation, turnover frequency calculations, mass activity normalization, and durability testing procedures. The adoption of unified benchmarking protocols and standardized reporting metrics will therefore be essential for establishing reliable structure–activity relationships and objectively evaluating catalyst performance across different studies.

### 7.5. Toward Sustainable and Economically Viable SACs

Future development should emphasize the replacement of scarce noble metals with earth-abundant elements such as Fe, Co, and Ni while maintaining high catalytic performance [207]. In parallel, sustainable catalyst design strategies, including the use of biomass-derived carbons and waste-derived support materials, can reduce production costs and improve environmental sustainability. The combination of low-cost materials, scalable synthesis, and long-term stability will ultimately determine the commercial viability of SACs for large-scale hydrogen production.

### 7.6. Toward Practical Electrolyzer Integration

Although the electrocatalytic performance of single-atom catalysts (SACs) has improved substantially, the vast majority of reported studies have been evaluated in laboratory-scale three-electrode configurations at current densities of approximately 10 mA cm^−2^. While such measurements provide valuable information regarding intrinsic catalytic activity, reaction kinetics, and mechanistic behavior, they do not necessarily predict catalyst performance under practical water electrolysis conditions. Commercial alkaline water electrolyzers (AWEs) and proton exchange membrane water electrolyzers (PEMWEs) typically operate at current densities of 1–2 A cm^−2^, where mass transport limitations, gas bubble accumulation, ohmic losses, and mechanical degradation become significantly more pronounced.

Bridging the gap between half-cell measurements and practical electrolyzer operation therefore remains a major challenge. Beyond achieving low overpotentials, SACs must maintain atomic dispersion and catalytic activity under prolonged high-current operation while resisting metal migration, aggregation, dissolution, and support corrosion. Furthermore, integration of SACs into membrane electrode assemblies (MEAs) introduces additional considerations that are rarely addressed in fundamental studies, including catalyst-layer architecture, catalyst loading, ionomer distribution, membrane compatibility, interfacial resistance, water management, and gas transport. Optimizing these parameters is essential because the intrinsic activity of the catalyst may not directly translate into improved device-level performance.

Another important limitation is that only a limited number of studies have evaluated SACs in complete electrolyzer devices under industrially relevant operating conditions. Most reports continue to focus on half-cell electrochemical measurements, making direct assessment of the practical advantages of SACs difficult. Recent demonstrations of SAC-based membrane electrode assemblies and full-cell electrolyzers have shown encouraging performance, indicating that atomically dispersed catalysts can remain active under practical operating conditions; however, these studies remain relatively scarce and require further validation under long-term continuous operation at commercially relevant current densities [208,209].

Future research should therefore place greater emphasis on translating SACs from fundamental electrocatalyst studies to practical electrolyzer systems. This requires standardized device-level testing protocols, systematic evaluation at industrially relevant current densities, extended durability assessments over thousands of operating hours, and close integration of catalyst design with MEA engineering. Combining operando characterization with realistic device testing will also be essential for understanding catalyst evolution under practical conditions and for establishing reliable structure–performance relationships. Such efforts will accelerate the commercialization of SAC-based electrocatalysts for large-scale hydrogen production.

Overall, addressing these challenges will require close integration of advanced synthesis, coordination engineering, operando characterization, theoretical modeling, data-driven catalyst design, and practical electrolyzer engineering. Progress in these areas will provide the foundation for developing highly efficient, durable, and economically viable SACs for practical water electrolysis.

## 8. Conclusions

Single-atom catalysts (SACs) have emerged as one of the most promising classes of electrocatalysts for water splitting owing to their catalyst-specific electronic structures determined by the metal center and local coordination environment. By bridging the advantages of homogeneous and heterogeneous catalysis, SACs have demonstrated remarkable HER and OER performance while substantially reducing the dependence on precious metals such as Pt, Ir, and Ru through maximized atomic utilization.

This review has highlighted the fundamental reaction mechanisms governing water electrolysis and discussed how rational catalyst design, including advanced synthesis strategies, support engineering, coordination environment regulation, and strong metal–support interactions, enables precise control over the activity, selectivity, and stability of isolated active sites. Recent advances in operando characterization and theoretical modeling have further deepened our understanding of the dynamic evolution of SACs during electrocatalysis, providing valuable insights for establishing reliable structure–activity relationships.

Despite these significant achievements, several challenges remain before SACs can be widely implemented in practical water electrolysis. Scalable synthesis, high metal loading while preserving atomic dispersion, long-term operational stability, comprehensive mechanistic understanding under realistic operating conditions, and validation in practical electrolyzer devices continue to be major research priorities. Furthermore, integrating machine learning with high-throughput computational screening, standardized evaluation protocols, and device-level testing is expected to accelerate the discovery and optimization of next-generation SACs.

Looking forward, the combination of earth-abundant transition metals, sustainable support materials, precise coordination engineering, and data-driven catalyst design is expected to further improve the efficiency, durability, and resource utilization of SACs. Continued advances in these areas, together with successful integration into practical electrolyzer systems, will facilitate the translation of SACs from laboratory-scale studies toward industrial water electrolysis and contribute to the development of efficient green hydrogen technologies.

## Data Availability

No new data were created or analyzed in this study. Data sharing is not applicable to this article.
